# Recombinant MVA-prime elicits neutralizing antibody responses by inducing antigen-specific B cells in the germinal center

**DOI:** 10.1038/s41541-020-00277-1

**Published:** 2021-01-25

**Authors:** Leila Eslamizar, Constantinos Petrovas, David J. Leggat, Kathryn Furr, Michelle L. Lifton, Gail Levine, Steven Ma, Christopher Fletez-Brant, Wesley Hoyland, Madhu Prabhakaran, Sandeep Narpala, Kristin Boswell, Takuya Yamamoto, Hua-Xin Liao, David Pickup, Elizabeth Ramsburg, Laura Sutherland, Adrian McDermott, Mario Roederer, David Montefiori, Richard A. Koup, Barton F. Haynes, Norman L. Letvin, Sampa Santra

**Affiliations:** 1 Center for Virology and Vaccine Research, Beth Israel Deaconess Medical Center, Harvard Medical School, Boston, MA USA; 2 Vaccine Research Center, NIAID, NIH, Bethesda, MD USA; 3 Foundation for the National Institutes of Health, Bethesda, MD USA; 4 Duke Human Vaccine Institute, Durham, NC USA; 5 Present Address: Integrative Toxicology, Nonclinical Drug Safety, Boehringer Ingelheim Pharmaceuticals, Inc., 175 Briar Ridge Road, Ridgefield, CT 06877 USA; 6 Present Address: Institute of Pathology, Department of Laboratory Medicine and Pathology, Lausanne University Hospital and Lausanne University, Lausanne, Switzerland

**Keywords:** Infectious diseases, Vaccines, Live attenuated vaccines

## Abstract

The RV144 HIV-1 vaccine trial has been the only clinical trial to date that has shown any degree of efficacy and associated with the presence of vaccine-elicited HIV-1 envelope-specific binding antibody and CD4+ T-cell responses. This trial also showed that a vector-prime protein boost combined vaccine strategy was better than when used alone. Here we have studied three different priming vectors—plasmid DNA, recombinant MVA, and recombinant VSV, all encoding clade C transmitted/founder Env 1086 C gp140, for priming three groups of six non-human primates each, followed by a protein boost with adjuvanted 1086 C gp120 protein. Our data showed that MVA-priming favors the development of higher antibody binding titers and neutralizing activity compared with other vectors. Analyses of the draining lymph nodes revealed that MVA-prime induced increased germinal center reactivity characterized by higher frequencies of germinal center (PNA^hi^) B cells, higher frequencies of antigen-specific B-cell responses as well as an increased frequency of the highly differentiated (ICOS^hi^CD150^lo^) Tfh-cell subset.

## Introduction

Vaccine development for HIV-1 has faced unprecedented challenges. To date, several HIV-1 vaccine concepts have been evaluated for efficacy in clinical trials^1–4^ and a new concept, that of mosaic HIV vaccines^5–7^, is currently being evaluated in the HVTN 705 and HVTN 706 clinical trials (NCT03060629 and NCT03964415)^8^. However, only one of these concepts, a canarypox ALVAC vector prime with an Env gp120 boost to date has shown modest efficacy in Thailand^9^. However, the same vaccination strategy of ALVAC vector prime using clade C immunogen conducted in South Africa, has failed to show any efficacy.^10^ These clinical trials have utilized different priming vectors including plasmid DNA^2,4^, recombinant adenovirus 5 (rAd5)^3^, and canarypox ALVAC^9^, whereas, the ongoing clinical trials are utilizing recombinant adenovirus 26 (rAd26) and modified vaccinia Ankara (MVA) as prime and boosting vectors, respectively^8^. Analyses of vaccine-induced immune responses in the past clinical trials have shown the role of priming vector and their roles in the induction of protective immunity^11^. Several preclinical and clinical studies have also assessed rAd26 and MVA as priming immunogen and have shown both of these vectors to be comparable^12^. However, MVA was shown to be less efficacious than rAd26 when used in the context of a boost in the preclinical study^12,13^. Therefore, selection of an appropriate priming vector is crucial for the development of a successful vaccine strategy. The immune responses that a priming vector must induce need to at least match the immune responses induced by ALVAC as till date that is the only modestly successful clinical trial.

The RV144 Thai trial showed that a priming immunization with ALVAC vector encoding subunit envelope immunogen that elicited helper CD4+ T-cell responses afforded modest protection against HIV-1 acquisition, whereas, the same immunogen failed to protect when administered alone^1^. In RV144, vaccinees received priming immunizations with rALVAC-HIV prior to boosting with AIDSVAX B/E gp120^14,15^. Immunizations with AIDSVAX B/E gp120 alone in the absence of priming in VAX003 failed to prevent HIV-1 infection^16^. Analyses of correlates of protection for RV144 showed that binding antibody responses against V1V2 region of envelope glycoprotein^11^ played an important role^17^. The ALVAC-HIV/AIDSVAX B/E vaccine regimen did not elicit broadly neutralizing antibodies (bNAbs)^18^. However, the vaccines elicited antibody-dependent cell-mediated cytotoxicity responses^19^, neutralizing antibodies to the tier 1 viruses and Env-specific CD4+ T-cell responses^14,20,21^. The development of pathogen and immunogen-specific B-cell responses requires the coordinated function of highly differentiated CD4+ T cells (Tfh) and B cells within germinal centers (GC)^22^.

Given the limited access to lymph nodes (LNs), several investigators have focused their analysis on circulating GC cell counterparts like circulating Tfh (cTfh)^23,24^. The predictive value of cTfh for vaccine-induced specific B-cell responses has been recently shown^25,26^. Furthermore, recent data have shown that optimal help by Tfh cells is critical for the development of bNAbs in a non-human primates (NHP) SHIV model^27^ possibly by regulating, among other factors, the metabolic program^28^ as well as the cell cycling/division of germinal center B cells in the dark zone^29,30^. Therefore, an optimal vaccine regimen must be able to generate robust antigen-specific CD4+ T-cell responses, including Tfh cells, which, in turn, would facilitate the development of humoral responses. A live vector prime/protein boost vaccination strategy has been widely used to generate such responses. However, the optimal priming vector for such an immunization is not known.

Here we have evaluated three priming vectors, plasmid DNA, recombinant MVA, and recombinant VSV with the same protein boost for their ability to prime for HIV-1 Env-specific antibody, CD4+ T and Tfh-cell responses. We found that recombinant MVA-prime optimally induced ICOS+ Tfh CD4+ T cells and elicited higher titers of antibody responses compared with plasmid DNA and rVSV.

## Results

### Priming with rMVA and rVSV elicited higher antibody-mediated neutralization activity compared with plasmid DNA

**Fig. 1:**
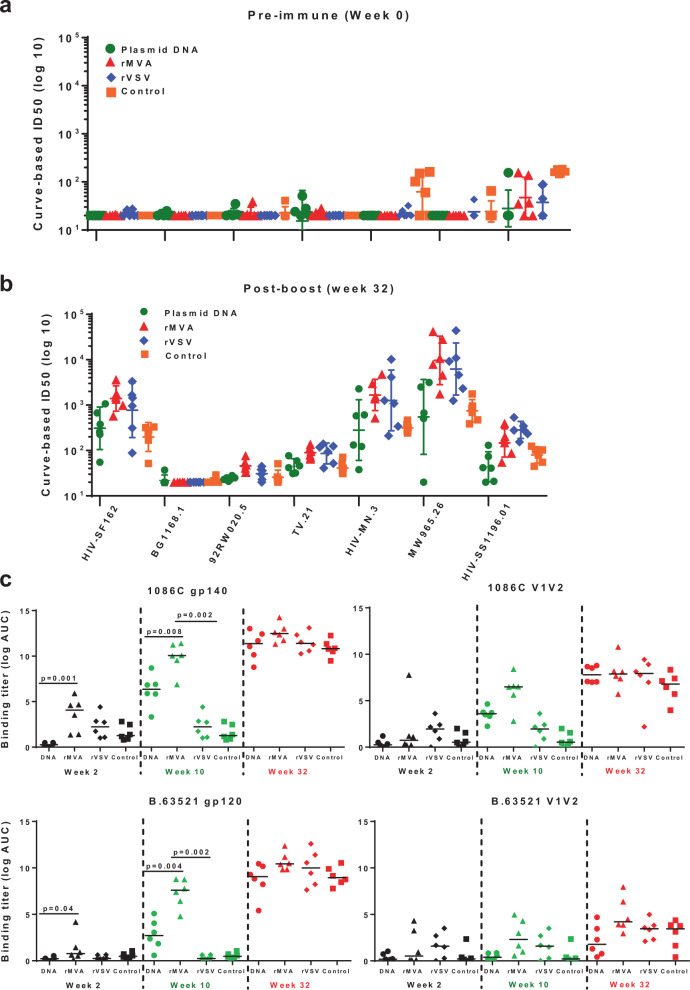
Antibody responses elicited by different priming immunizations. Neutralizing antibody responses as measured by TZM-bl cell-based assay at pre-immune, week 0 (**a**) and post-boost, week 32 (**b**) time points. TZM-bl cell-based neutralization assays were performed against both Tier 1 and Tier 2 viruses. Curve-based log ID50 values for pre-immunization and post-protein boost time points are shown for plasmid DNA (green circles), rMVA (red triangles), and rVSV (lavender diamonds), and protein only control (orange squares) groups. ID50 titers against seven different viruses, HIV-1 SF162, BG1168.1, 92RW020.5, TV.21, HIV-MN.3, MW965.26, and HIV-SS1196.01 are shown with geometric mean with 95% CI of the six monkeys per group. **c** Binding antibody titers elicited by different priming immunizations. ELISA binding titers (log AUC) of plasma against autologous (upper panels) and heterologous (lower panels) antigens from individual monkeys in four vaccine groups are shown at Week 2 (black symbols), Week 10 (green symbols), and post-boost (red symbols) time points. The lines indicate the median of the six monkeys per group.

Neutralizing antibody responses from all four groups of vaccinated monkeys were assessed against a panel of tier 1 and tier 2 viruses using both TZM-bl and A3R5 cell-based assays. Two weeks following the protein boost, neutralizing antibody responses against the HIV MW965.26 clade C, MN.3 clade B, and SF162 clade B were significantly higher in monkeys that were primed with rMVA or rVSV compared with either plasmid DNA (*p* = 0.0087, *p* = 0.0649 and *p* = 0.0152 against HIV MW965.26 clade C, MN.3 clade B and SF162 clade B, respectively) or protein only (*p* = 0.002, *p* = 0.004, and *p* = 0.002 against HIV MW965.26 clade C, MN.3 clade B, and SF162 clade B, respectively) groups (Fig. 1a, b). Although not significant, rMVA elicited higher neutralization titers for several viruses (HIV-SF162, 92RW020.5, TV.21, HIV-MN, and MW965.26) compared with rVSV that had the highest neutralization titers against HIV 1196.01, a tier 1B neutralizer (Fig. 1b). Neutralizing antibodies against tier 2 virus TV1.21 was weak in magnitude in all four groups of animals. However, monkeys in both rMVA- and rVSV-primed groups had higher titers of neutralization against this virus compared with other two groups. No neutralization was detected against 92RW020.5 (tier 1) and BG1168.1 (tier 2) viruses. An A3R5 cell-based assay was used to assess vaccine-elicited tier 2 neutralizing antibodies in these cohort of monkey, as it is known to be more sensitive than TZM-bl cell-based assay for detecting neutralizing antibodies. We did not detect any neutralization titers against Ce1176 A3, Du151.2 and CE2010 F5 viruses (Supplementary Figure 1, panels a-c). Autologous neutralizing antibodies against C.1086 virus were elicited in all three groups of monkeys that received priming immunizations with either plasmid DNA, rMVA, or rVSV. The magnitude of autologous neutralization was lower in the monkeys that were immunized only with protein without any vector priming (Supplementary Figure 1d). As expected, neutralization titers against TV1.21 were higher in the A3R5 assay than in TZM-bl cells (Supplementary Figure 1e). Both assays confirmed that rMVA and rVSV priming elicited the highest levels of neutralizing activity with rMVA been more potent for some of the tested viruses.

### rMVA prime elicited highest level of autologous and heterologous binding antibodies

Next, the binding antibody titers in all groups of the vaccinated monkeys against both clades B and clade C Env antigens was assessed. The panel used to assess vaccine-elicited binding antibody responses consisted of the following immunogens: C.1086 gp120, C.1086 gp140, V1V2 of 1086 C (C.1086 V1V2 tags, C.1086 V1V2 N156Q), B.63521 gp120, B.63521 V1V2 tags, and B.63521 V1V2 N156Q N160Q. Two weeks after the first priming immunizations among all three groups of monkeys that received vector priming, autologous and heterologous binding antibody titers were significantly higher in rMVA-primed group compared with the group primed with plasmid DNA (*p* = 0.001 and *p* = 0.04, respectively). Recombinant MVA-primed monkeys also had higher titer compared with rVSV-primed group. Titers for both autologous and heterologous binding antibodies were assessed 2 weeks after final priming immunizations at week 10. At this point, monkeys in plasmid DNA group received a total of three (weeks 0, 4, and 8) and rMVA group received two priming immunizations (week 0 and 8), whereas, rVSV monkeys were primed once on week 0. The priming regimen for the three vectors were determined based on previous works using these vectors.^31–33^ Post-prime titers of binding autologous, as well as heterologous antigens, were significantly the highest in the rMVA-primed group among all vector-primed groups (Fig. 1c). Antibody titers against the V1/V2 region of either C.1086 or clade B immunogen B.63521 were low. Plasma samples from all groups of monkeys were tested again at 2 weeks post-protein boost. As shown in Fig. 1c (week 32), irrespective of the type of priming immunizations, the titers of both autologous and heterologous binding antibodies were very similar across all groups.

### Plasmid DNA prime induced higher Th1 type cytokines than priming with either rMVA or rVSV

**Fig. 2:**
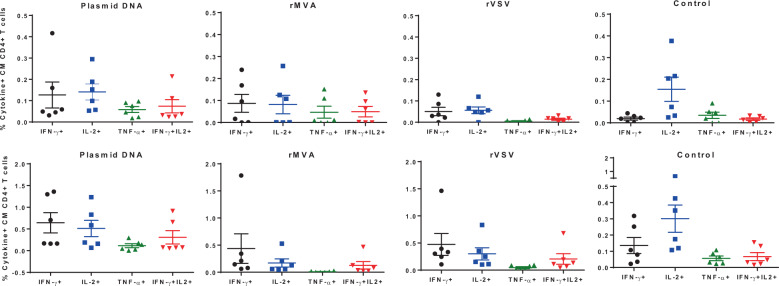
Cytokine secretion profile of the PBMC of the vaccinated monkeys as measured by ICS assay. Percentages of post-prime (upper panels) and post-protein boost (lower panels) cytokine-secreting CD4+ central/transitional memory T cells (T_CM/TM_) were quantified in all four groups of monkeys following stimulation with C.1086 Env peptide pool. Percentages of IFN-γ (black circles), IL-2 (blue squares), TNF-α (green triangles), both IFN-γ/IL-2 (red inverted triangles) secreting T_CM/TM_ CD4+ T cells are shown for individual monkeys for each vaccine group with mean and SEM.

Cytokine production by T cells upon antigen stimulation is a quantitative way to measure T-cell functionality. Post-prime (upper panel) and post-boost (lower panel) cytokine (IFN-γ IL-2, TNF-α)-secreting central/transitional memory T cells (T_CM/TM_) in the peripheral blood were quantified in all groups of monkeys (Fig. 2). For the post priming responses (Fig. 2, upper panel), plasmid DNA induced the highest levels of IFN-γ+ or IL-2+ CD4+ T-cell responses, followed by the rMVA and rVSV-induced responses (Fig. 2). Regarding the protein boost induced responses, a similar pattern was observed (Fig. 2, lower panel). Interestingly, plasmid DNA prime was able to induce both IFN-γ+ and IL-2+ significantly higher (*p* < 0.05; Kruskal–Wallis test) compared with post priming levels while rMVA induced selectively the IFN-γ+ CD4+ T-cell responses (Fig. 2, lower panel). This observation was also confirmed by IFN-γ ELISpot assay where the number of IFN-γ-producing spot-forming cells were significantly higher in monkeys in those two groups. A low frequency of IFN-γ+ IL-2+ CD4+ T cells across the groups was found pre and post boosting (Fig. 2). Therefore, different vectors induce different patterns of circulating cytokine-secreting CD4+ T-cell responses.

### Antigen-specific memory CD4+ T cells in the periphery of rMVA-primed monkeys show a unique transcriptome profile

**Fig. 3:**
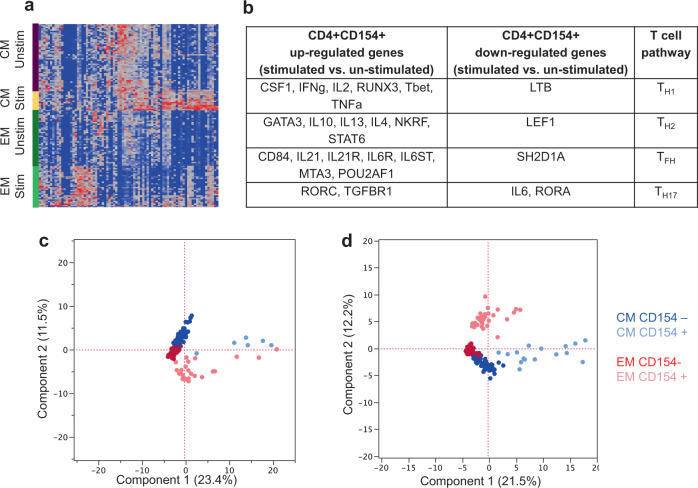
Distinct gene expression profile in post vaccination sorted antigen-specific T_EM_ and T_CM_ populations. **a** Gene expression heat map identifying uniquely uregulated genes within T_CM_ and T_EM_ compartments for all 24 rhesus macaques (red indicates upregulation). **b** Gene expression profile between 1086.C ENV peptide stimulated and unstimulated cells describing up- and downregulated genes of CD4+ CD154+ populations. **c**, **d** The SVM-RFE (Support Vector Machine with Recursive Feature Elimination) transcriptional profiling is shown for T_CM_ and T_EM_ compartments based on expression of CD154 in **c** post prime and **d** post boost time points. Both T_CM_ and T_EM_ cells could be readily discriminated based on CD154 expression.

For an in-depth analysis of transcriptional signatures of vaccine-elicited memory CD4+ T-cell populations, we measured the expression signals of 96 genes in circulating sorted T_CM_ and T_EM_ cells using the Fluidigm technology. Peripheral blood mononuclear cells (PBMC) from all 24 rhesus macaques were stimulated with pooled peptides spanning 1086.C gp120 and antigen-specific CD4+ CD154+ T_CM_ and T_EM_ cells were sorted as described in the Methods section. T_CM_ cells were defined as live CD3+ CD4+ CD95+ CD28+ CCR7+ T cells and T_EM_ cells were gated as CD3+ CD4+ CD95+ CD28− CCR7− T cells. Distinct gene expression profiles were observed with or without antigen-stimulated CD4+ populations showing vaccination overall resulted in the activation of both types of memory T cells (Fig. 3a). SVM-RFE (Support Vector Machine with Recursive Feature Elimination) was used to analyze the gene expression of CD4+ CD154+ T cells among all groups. The gene expression profile between peptide stimulated cells and unstimulated cells showed differential expression of several genes important for the development of multiple T-cell lineages (Th1, Th2, Tfh, Th17), regardless of vaccine regimen (Fig. 3b), showing vaccination-induced broad T-cell responses. Furthermore, a distinct profile was found between antigen-specific (CD154+) and bulk CD4+ T cells both in T_CM_ and T_EM_ compartments at post-prime (Fig. 3c) and post-boost (Fig. 3d) time points. These differences show priming and boosting have differential effects on the T_CM_ and T_EM_ compartments which may be important for the development of the broad T-cell responses observed. Because rMVA immunization provided the best neutralization titers, the gene expression of cells from rMVA group (post-boost time point) was compared with the other groups (Supplementary Table 2). Our analyses revealed an upregulation of genes such as IL-6ST, GP130, Bcl-6 and IL-4 (Tfh factors), IL-10, IL-13 (Th2), and IL-17 (inflammatory-Th17) selectively in CD4+ CD154+ T cells from the rMVA group (Supplementary Table 2). Therefore, our gene analyses data support the neutralizing and antibody titers data further suggesting that rMVA immunization favors the development of neutralizing antibodies compared with other vaccine platforms tested in this study.

### Vector priming induces LN immune dynamics favoring the development of vaccine-induced B-cell responses

**Fig. 4:**
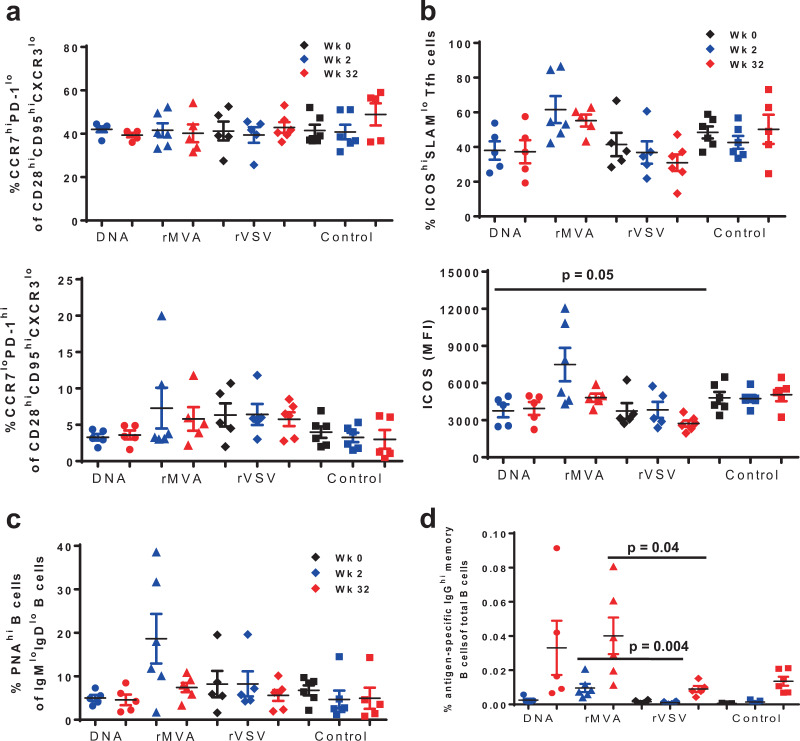
Increased expression of ICOS on Tfh CD4+ T cells is associated with induction of Env-specific B-cell responses after priming with rMVA. In each panel, black symbols denote pre-immunization (Week 0), blue symbols post-prime (Week 2) and red symbols show post-boost measurements. Plasmid DNA group is represented by using circles, rMVA by triangles, rVSV by diamonds, and control group by squares. Bars depict SEM. **a** Both upper and lower panel show the percentages of Tfh CD4+ T cells based on the expression of CCR7 and PD-1 in lymph node-derived lymphocytes. **b** Upper panel shows the percentages of ICOS^hi^SLAM^lo^ Tfh CD4+ T cells for individual monkeys from each vaccine group at weeks 0, 2, and 32. Lower panel shows the expression of ICOS per Tfh CD4+ T cell—based on Mean Fluorescence Intensity (MFI). **c** Dot plots showing accumulated data of relative frequencies (%) of bulk germinal center memory B cells in lymph node (LN) tissues at the indicated time points as judged by PNA staining—memory B cells in LN tissues obtained at the indicated time points. **d** Immunogen-specific B cells responses were detected by using a specific Env probe in bulk memory B cells from LN tissues. The relative frequencies are shown as a percentage of total B cells. All LN samples were analyzed in one experiment. Non-parametric Mann–Whitney *U* test was used for the statistical analysis and mean and SEM are shown for each vaccine group.

Follicular helper CD4+ T cells (Tfh) represent a population with unique phenotype, characterized by high expression of CXCR4, CXCR5, PD-1, ICOS, and low expression of CCR7, CCR6, and CD127, and localization in follicular areas and particularly GCs^34–36^. Circulating counterparts of Tfh (cTfh) have been described based on the expression of receptors like CXCR5, PD-1, ICOS^24^. Thus in this study, the expression of several molecules that are important for Tfh function were analyzed on CD4+ T_CM_ cells (CD3+ CD4+ CD28^hi^CD95^hi^) that had low expression of CXCR3, a marker of Th1-like LN Tfh, functionally distinct from “classic” germinal center Tfh cells.^37^ Regardless of vaccine modality, the majority of CD4+ T_CM_ in LN showed high expression of CCR7 and low expression of PD-1 (Fig. 4a, upper panel). The relative frequency of bulk GC-Tfh (CCR7^low^PD-1^high^) was significantly lower in comparison with CCR7^high^PD-1^low^ population and it did not change after prime or boost immunization within each group (Fig. 4a, lower panel). We have previously showed that NHP Tfh cells express a high ICOS phenotype while the expression of SLAM (CD150) can further define Tfh subpopulations with different capacity for in vivo proliferation^36^. Furthermore, ICOS^high^SLAM^low^ was the Tfh population with higher capacity for IL-4 production^36^ a cytokine critical for the GC B-cell development^38^. Therefore, we sought to analyze the expression of these receptors on the Tfh cells from different vaccinations. The rMVA group had a significantly higher frequency of ICOS^high^SLAMl^ow^ Tfh cells (*p* = 0.0087) even compared with rVSV group (Fig. 4b, upper panel). In line with this, the expression of ICOS per cell, judged by the mean fluorescence intensity-MFI, was also significantly higher in the rMVA group at 2-week post-prime compared with the Tfh cells in other groups (Fig. 4b, lower panel). Furthermore, a trend for higher expression of CXCR4 per cell was found on ICOS^high^SLAM^low^ Tfh in rMVA compared with other groups (Supplementary Figure 2). Although LN cells from pre-vaccinated animals were not available for the rMVA group owing to technical difficulties in LN collection, our analysis showed no difference between the groups when the bulk non-Tfh and Tfh-cell populations were investigated (Fig. 4a), indicating that the higher frequency of ICOS^high^SLAM^low^ Tfh cells, as well as the expression of ICOS per cell, are possibly related to rMVA vaccination.

The frequency of immunogen-specific Tfh CD4+ T cells was analyzed following in vitro stimulation with peptide pool spanning the entire 1086.C gp120 and detection of CD154 mobilization. The group primed with rVSV showed the highest frequency of antigen-specific Tfh CD4+ T cells followed by the rMVA group, particularly after boosting (Supplementary Figure 3a). A similar profile was found when antigen-specific CXCR5^hi^ cTfh cells were analyzed (Supplementary Figure 3b). We further analyzed the frequency of IFN-γ responses in the antigen-specific Tfhs. Again, rVSV was the group characterized by the highest frequency of Tfh cells able to produce IFN-γ (Supplementary Figure 3c). No differences were found when the production of IL-4 was analyzed in all groups. Overall, our data suggest that rMVA prime induces germinal center immune dynamics favoring the development of vaccine-induced B-cell responses even after one priming immunization.

### B-cell development inside the germinal center is facilitated by rMVA prime

**Fig. 5:**
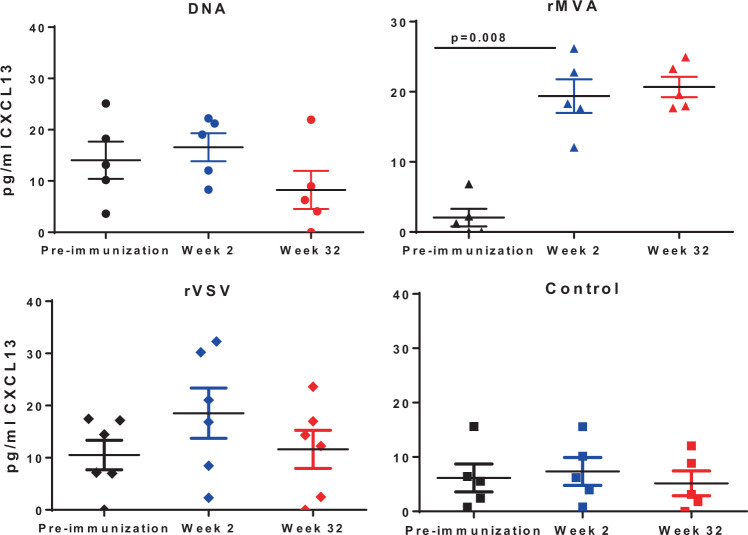
rMVA prime monkeys have highest CXCL13 levels in their plasma. Plasma samples from pre-immunization (black symbols), Week 2 (blue symbols), and Week 32 (red symbols) from all monkeys in four vaccine groups were assessed for CXCL13 levels by ELISA assay following manufacturer’s protocol (R&D Systems). Level of CXCL13 (pg/ml) was derived from the standard curve generated from the OD values at 570 nm. Mean and SEM are shown for each vaccine group.

Next, we investigated the dynamics of B cells in LNs obtained at pre-immunization, post-prime and post-boost time points from all groups of animals. Similar relative frequencies of bulk memory (IgD^lo^IgM^lo^) B cells were found across the animal groups tested. A similar profile was found when the expression of surface IgG on memory B cells was analyzed (Supplementary Figure 4). Using PNA, a marker of GC B cells^36^, we found a significant induction of the GC B cells post priming (Week 2) selectively in the rMVA group (Fig. 4c). Furthermore, this induction was associated with increased frequency of immunogen-specific B-cell responses as measured by the binding of a gp120-specific probe post-prime in the rMVA-primed group (Fig. 4d). To confirm the GC activity in monkeys primed with rMVA, we measured plasma CXCL13 levels, a surrogate of GC activity^39^, in all four groups of monkeys. Plasma CXCL13 levels were measured in all four groups of monkeys at pre-immunization, post-1st prime (week 2) and post-boost time points (week 32). As shown in Fig. 5, two weeks post-prime at week 2 both rVSV- (twofold increase) and rMVA-primed (15-fold increase) monkeys had higher plasma CXCL13 levels than plasmid DNA and protein control group. Monkeys primed with rMVA had a significant increase in plasma CXCL13 level two weeks post-first prime at week 2 (*p* = 0.008). However, the post-boost plasma CXCL13 level in rVSV-prime group contracted to the pre-immunization level whereas, in rMVA-primed monkeys the post-boost level remained at the post-prime level indicating GC activity as a result of priming with rMVA.

## Discussion

We have investigated biological factors associated with the development of B-cell responses in NHPs vaccinated with different vectors/carriers encoding a clade C HIV-1 Env. The development of antigen-specific antibodies requires the coordinated interaction of highly differentiated CD4+ T and B cells within the germinal center. The quality of provided help by Tfh cells directs the fate of dividing B cells within the dark zone of the germinal center and the development of immunoglobulin mutations directly related to the neutralization potential of antigen-specific antibodies.^22^ Analysis of plasma Env-specific antibodies showed that rMVA was the regime inducing the highest titers compared with other vectors for both autologous and heterologous Env strains. Furthermore, rMVA boost induced similar or higher neutralizing activity compared with other vectors. Post-boost induced humoral responses can result from the reactivation of memory responses formed during the priming and possibly the generation of new responses during the boosting phase. The titers of the specific antibodies after priming indicate that rMVA generates higher antigen-specific memory B-cell responses. However, it should be pointed out that the vaccinated groups did not receive the same number of priming doses, which can affect the outcome of the immune dynamics under investigation. Despite the lower titers post priming, rVSV boosting led to the highest fold change of antibody titers compared with other vectors. Therefore, we can hypothesize that the quality and/or quantity of rVSV-induced memory Tfh/B cells during priming is different compared with the other vectors. The vaccination scheme that was used in the current study could be, at least in part, a critical determinant for these differences. Further investigation is needed to determine whether the different neutralization activity observed among the vectors, reflects different affinity/avidity of relevant antibodies between the groups and/or different elicited epitope antibody specificities.

Analysis of circulating antigen-specific (cytokine+) CD4+ T cells revealed that plasmid DNA vaccination, associated with the lowest neutralization activity, induced the higher frequency of IFN-γ+ and IL-2+ CD4+ T cells both at post-boost and post-prime time points. Our data indicate a dissociation between the elicitation of classic circulating Th1 CD4+ responses and the generation of neutralizing antibodies. That was more evident in the DNA vaccinated animals indicating that plasmid DNA elicited vaccine responses are more skewed towards a Th1 phenotype, at least in this experimental settings. Compared with rMVA, vaccination with rVSV induced higher levels of CD154+ IFN-γ+ Tfh cells in the LN both at week 2 and week 32. This profile was also associated with higher circulating IFN-γ+ CD4 T cells at week 32 in rVSV compared with rMVA vaccinated animals. Tfh represent a highly heterogeneous group of cells with presumably different functions^38^. Previous work has shown that CXCR3 + (Th1 like) Tfh cells could support B-cell responses, although the impact of IFN-γ in the in vitro IgG production by B cells was modest and significantly lower compared with the impact of IL-21^37^. Furthermore, a recent report showed that IL-21 and IFN-γ were produced by different human tonsillar Tfh-cell subsets^40^. Therefore, the co-expression of IFN-γ with other mediators that could impact the B-cell responses (e.g., IL-21, IL-4) is critical in order to better understand the role of the IFN-γ+ Tfh cells. In our experimental settings the prevalence of IFN-γ+ Tfh cells was not correlated with better antigen-specific LN B-cell responses in the rVSV group. Whether this is owing to a particular cytokine production profile of Tfh cells (our gene sequencing analysis suggests a higher IL-4 expression in cTfh cells induced by rMVA, a vector associated with a lower frequency of IFN-γ+ Tfh cells) or the altered activity of other mechanisms regulated by IFN-γ (e.g., production of BAFF^41^) needs further investigation.

Upon stimulation, distinct T-cell pathways for Th1, Th2, Tfh, and Th17 cells could be identified by up- and downregulation of selected genes that are important for different T-cell development regardless of vaccine regimen. Our data highlight a select signature of genes capable of distinguishing T_CM_ and T_EM_ populations. T_CM_ and T_EM_ populations differed in CD154 (CD40L) expression when comparing post-prime and post-boost samples. However, CD154 expression is not independently sufficient to distinguish these populations. Direct comparison of post-prime and post-boost populations demonstrated an increase in T_CM_ CD154+ cells post-boost, suggesting the immune system may yield an improved anamnestic response to subsequent exposure to 1086.C Env. Still, rMVA induced a pattern closer to Tfh further supporting rMVA prime in line with its profile of cytokine-induced CD4 T-cell responses and antigen-specific neutralization activity.

In addition to circulating cells, analysis of LN-derived cells, when available, showed that rMVA priming was associated with the highest levels of germinal center (PNA^hi^) B cells and ICOS^hi^SLAM^lo^ Tfh cells, which are characterized by the highest capacity for IL-4 production^36^, a cytokine critical for the maturation/switching of B cells^42^. This profile is concordant with the higher autologous and heterologous antibody-binding titers found after priming with rMVA compared with other groups. Furthermore, the increased post-prime percentages of GC B cells and antigen-specific B cells found in rMVA animals are in line with the high expression of ICOS on T_fh_ cells post-prime in this group of animals. rMVA vaccination induced the higher levels of ICOS expression on Tfh cells, a receptor possibly mediating the mutual regulation between Tfh and B cells^43^. Supporting the favorable activity of rMVA vaccination for the development of humoral responses is the induction of significantly higher levels of bulk GC B cells and antigen-specific IgG^hi^ B cells in the follicles even at week 2 post priming. Therefore, the direct comparison between the groups at week 2 (after one priming dose) indicates that rMVA vaccination initiates an early germinal center reactivity favoring the development of antigen-specific antibodies compared with other regimens. Circulating CXCL13, the ligand for CXCR5 and a critical regulator for the trafficking of CD4+ T cells into the germinal center^44^, has been proposed as a surrogate for GC reactivity associated with vaccine outcome^39^. We found that only rMVA was able to continuously induce and maintain higher levels of CXCL13 compared with other vectors, in line with the higher frequencies of ICOS^hi^SLAM^lo^ Tfh cells post boosting/post priming. This is in line with previous reports showing that vaccination of mice with rMVA was able to induce tertiary lymphoid structures enriched in CXCL13 and capable of supporting T-cell priming.^45,46^ Overall, our data show that (i) different vector-based vaccines are associated with differential development of germinal center reactivity and generation of neutralizing antibody responses and (ii) indicate that the germinal center dynamics during the primary phase is a critical factor for the development of the humoral responses after boost. The molecular and cellular mechanisms responsible for this outcome are not known and need further investigation. The use of the NHP model and relevant vaccination strategies could provide guidance for the development of “effective” germinal center reactivity and possible targets for in vivo manipulation aiming to further strengthen the production of bNAbs.

## Methods

### Generation of plasmid DNA, rMVA, and rVSV expressing C.1086 *env* gene

DNA plasmid containing HIV-1 C.1086 *env* gene were generated by cloning the virus cDNA into the pVR1012 vector as previously described^47,48^. The codon-optimized plasmid for HIV-1 C.1086 gp120 monomer was commercially synthesized (Genewiz, Inc) and cloned into the pCD4+ NA3.1+ expression vector (Invitrogen). Purification method is described in Fouda et al. (2013). DH5α bacteria containing the pCD4+ N31.1+ plasmid expression vector containing the HIV-1 C.1086 gp120 envelope sequenes were grown up and purified using plasmid purification kits (Qiagen). Recombinant MVA expressing HIV-1C.1086 *env* gene was generated as described^48^. In brief, the HIV Env C.1086 gp140 protein corresponding to residues 1–669, with modifications of E489R and E497R to mutate the gp120-gp41 cleavage site, was expressed in an MVA virus. This gene, under the control of a poxvirus early/late promoter, was placed into the genome of MVA A681 virus by means of the insertion plasmid p2614. After transfection of insertion plasmid DNA into cells infected with A681 virus, recombinant viruses capable of replication in RK13 cells were selected (containing the *gp140* gene, the *GUS* gene, and the *K1L* gene) and then passaged in BHK21 cells, without selective pressure for the *K1L* gene. A virus lacking the marker cassette but containing the *gp140* gene was isolated. Recombinant VSV expressing HIV-1 C.1086 *env* gene was generated following methods described previously^49^. To obtain plasmids that could be used to recover rVSV expressing the HIV Env from the fifth position in the VSV genome, the *env* gene sequences from HIV-1C.1086 were PCR-amplified from plasmids. The forward primer introduced a Xho I site upstream of the coding sequence and the reverse primer introduced an Nhe I site. PCR products were digested with Xho I and Nhe I, purified, and ligated into the pVSVXN2 vector that had been digested with the same enzymes (VSV cloning vectors provided by Dr. John Rose, Yale University). Plasmids were recovered after transformation of *Escherichia coli* and purified using a Maxi kit (QIAGEN) and the inserted sequences verified (Duke DNA Analysis Facility). Recombinant virus was recovered from the pVSVXN2 1086 C plasmid as previously described^50^.

### NHP study design

Monkeys were housed at the New England Primate Research Center (Southborough, MA) and were maintained in accordance with the recommendations of the Association for Assessment and Accreditation of Laboratory Animal Care International Standards and with the recommendations in the Guide for the Care and Use of Laboratory Macaques of the United States—National Institutes of Health. The Institutional Animal Use and Care Committee of Harvard Medical School approved these experiments (Protocol # NLL03503). Twenty four Indian-origin rhesus (*Macaca mulatta*) monkeys were distributed in four experimental groups (*n* = 6 per group). Monkeys in group 1 were primed with 5 mg plasmid DNA at weeks 0, 4, and 8. Monkeys in group 2 were primed with 10^9^ pfu of rMVA at weeks 0 and 8, and monkeys in group 3 were primed with 10^8^ pfu of rVSV (Indiana strain) at week 0. All three of the vaccine vectors encoded HIV-1 clade C transmitted/founder Env C.1086 gp140^17^. At week 30 all monkeys in groups 1–3 were boosted with 100 μg of HIV-1 C.1086 gp120 protein formulated in MF59 adjuvant. Monkeys in group 4 were immunized with C.1086 gp120 protein in MF59 at weeks 0 and 10 without any vector prime (Supplementary Table 1). In addition to pre- and post-immunization blood draws, LN biopsies were collected at 2 weeks post-protein boost.

### Intracellular cytokine staining assay

PBMC were incubated in Roswell Park Memorial Institute (RPMI) supplemented with 10% fetal bovine serum, 100 U/ml penicillin, and 100 μg/ml streptomycin (RPMI-10) at 37 °C in a 5% CO_2_ environment for 6 h in the presence of either dimethyl sulfoxide (unstimulated), pool of overlapping C.1086 Env peptides or 0.4 μg/well staphylococcal enterotoxin B (Sigma) as positive control. The peptide pool spanning the entire HIV-1 Env C.1086 comprised of 15 amino acid peptides overlapping by 11 amino acids. All wells contained 2 μM of a protein transport inhibitor, monensin (GolgiStop; BD Biosciences) and 1 μg/ml anti-human CD49d antibody (BD Biosciences). The cultured cells were then stained with the following mAbs: anti-CD4 APC-H7 (BD Biosciences), anti-CD95 PE (BD Biosciences), anti-CD28 PerCP-Cy5.5 (BD Biosciences), and anti-CCR7 FITC (R&D Systems). After fixation and permeabilization with Cytofix/Cytoperm solution (BD Biosciences), the cells were stained with anti-IFNγ PECy7 (BD Biosciences), anti-TNFα AF700 (BD Biosciences), anti-IL-2 APC (BD Biosciences), anti-CD3 Pacific Blue (BD Biosciences), and anti-CD69 ECD (Beckman Coulter), and fixed with 1% formaldehyde. Samples were collected on a LSRII flow cytometer (BD Biosciences) and analyzed by using FlowJo software (TreeStar). Approximately 250,000–900,000 events were collected per sample. Doublets were excluded from analysis by gating singlets in forward scatter-area versus forward scatter-height analysis. Three subgroups of CD4+ T cells were defined as (i) naive (CD3+ CD4+ CD28+ CD95-), (ii) central memory (CD3+ CD4+ CD28+ CD95+), and (iii) effector memory (CD3+ CD4+ CD28− CD95+) (Gating strategy in Supplementary Figure 5a). Functional analyses were performed by viewing the expression of each cytokine against the early activation marker CD69.

### Tfh-cell phenotyping and antigen-specific Tfh analysis

One to two million lymphocytes from axillary LN were stained with viability dye (Aqua amine viability dye, Invitrogen) followed by surface staining using the following antibodies: CD3-Cy7APC (clone SP34-2, BD Biosciences), CD4-Cy55PE (clone S3.5, Invitrogen), CD28-ECD (clone Tp44, Beckman Coulter), CD95-Cy5PE (clone DX-2, BD Biosciences), CCR7-Alx700 (clone 150503, BD Biosciences), PD-1 BV421 (clone EH12.2H7, Biolegend), ICOS-Alexa647 (clone C398.4 A, Biolegend), CD150-PE (clone A12, Biolegend), CXCR4-Cy7PE (clone 12G5, Biolegend), CD20-BV570 (clone 2H7, Biolegend) and CXCR3-FITC (clone CXCR3-173, eBiosciences).

To detect antigen-specific Tfh cells, 3–4 × 10^6^ LN cells were incubated in 1 ml of RPMI-10% fetal bovine serum-containing monensin (0.7 μg/ml; BD Biosciences) and brefeldin A (10 μg/ml; Sigma-Aldrich) in the absence or presence of peptides (HIV Env C) for 6–7 h. Cells were washed and surface stained with Aqua and titrated antibodies: CD4-BV605 (clone L200, BD Biosciences), CD8-BV785 (clone RPA-T8, Biolegend), CD28-ECD (Beckman Coulter), CD95-Cy5PE (clone DX-2, BD), CXCR5-efluor710perCp (clone MU5UBEE, eBioscience), CCR7-Alx700 (clone G043H7, Biolegend) and PD-1-BV650 (clone EH12.2H7, Biolegend). After fix/permeabilization, cells were stained with CD3-Cy7APC, CD154-PE (clone TRAP1, BD Biosciences), IL-4-Cy7PE (clone 8D4-8, BD Biosciences), IL-21-Alx647 (clone 3A3-N2.1, BD Biosciences) and IFN-γ-FITC (clone B27, BD Biosciences), incubated for 30 min, washed and fixed in 1% paraformaldehyde (gating strategy in Supplementary Figure 5c). Events were collected on a modified LSRII flow cytometer (BD Immunocytometry Systems). Electronic compensation was performed with antibody capture beads (BD Biosciences) stained separately with antibodies used in assay. Data were analyzed using FlowJo Version 9.6 (TreeStar, Ashland, OR).

### Phenotyping of lymph node B cells

Lymph node-derived lymphocytes were stained with Aqua and the following titrated antibodies: CD3-Cy7APC, CD20-BV570, IgD-PE (Southern Biotech, cat no.2030-02), IgM-Cy5PE (clone G20-127, BD Biosciences), IgG-APC (clone G18-145, BD Biosciences), CD27-BV605 (clone O323, Biolegend), PNA-PE (GeneTex), CD86-FITC (IT2.2, BD Biosciences) and CXCR4-Cy7PE. In all, 2–3 × 10^6^ cells were stained with Aqua and biotinylated Env gp120 probe on ice for 30 min. Following a staining step with streptavidin, cells were surface-stained with antibodies and peanut agglutinin (PNA). The staining step was followed by washing and fixation using 1% paraformaldehyde (gating strategy in Supplementary Figure 5d).

### Staining and sorting of CD4+ CD154+ T cells for Fluidigm experiments

PBMCs were stimulated overnight with peptides spanning 1086.C Env at a final concentration of 1 μg/ml along with CD154 Cy5PE as a co-stimulant (BD Biosciences; 555701). After stimulation, the cells were washed and surface stained with the following antibodies for 20 min at 4 C: anti-CD4-Cy55PE (BD Biosciences; MHCD0418), anti-CD3-Cy7APC (BD Biosciences), anti-CD14 BV510 (Biolegend; 301841), anti-CD8 BV510 (Biolegend; 301047), anti-CD95 APC (BD Biosciences; 558814), anti-CD28-ECD (Beckman Coulter; 6607111), anti-CD19 BV510 (Biolegend; 302242), anti-CCR7 BV421 (Biolegend; 353208), anti-CXCR3 Ax700 (BD Biosciences; 561320), anti-PD-1 BV605 (Biolegend; 329923), anti-CXCR5 Ax488 (NIH NHP reagent resource), anti-CCR6 PE (Biolegend; 353410), ICOS Cy7PE (BD Biosciences; 313520), and Live/Dead Fixable Aqua dead cell stain (Life Technologies).

After staining the cells were washed twice with FACS wash and passed through BD Falcon cell strainer cap before performing the sort. Three populations (CD4+ CD28+ CD95+ central memory, CD4+ CD28-CD95+ effector memory, CD4+ CD28+/−,CD95+ total memory) of 25 live CD3+ CD4+ CD154+ cells/well were sorted in duplicate using BD FACSARIA into 96-well plates (Gating strategy in Supplementary Figure 5b). The wells of the 96-well plate had RT-Pre Amplification Reaction Mix, containing CellsDirect One-shot qRT-PCR buffer at 2× concentration, Superscript III reverse transcriptase and Platinum Taq polymerase (Life Tech; 11732-088), SuperaseIN RNase inhibitor (Life Tech; AM2696) and a mixture of the Taqman ABI primers and probes (Supplementary Table 3) specific for the transcript of interest at 0.2× concentration. Once the sort was completed, 96-well plates with the samples were briefly centrifuged and the PCR step was performed. The conditions for the PCR step were 50 °C for 15 min for the reverse transcription (RT), 95 °C for 2 min for inactivation of RT enzyme and followed by 18 cycles of 95 °C for 15 sec and 60 °C for 4 min. After the end of the PCR step, the cDNA sample was stored at −80 °C until further use. When ready for the analysis of the cDNA samples, the samples were thawed, vortexed, centrifuged, and further diluted (1:5) in DNA suspension buffer (Teknova; T0223). The diluted cDNA samples were mixed into a mixture of mild detergent loading solution (Fluidigm; 100–7610) and TaqMan Universal PCR Master Mix (Life Tech; 4304437). The 20× TaqMan ABI primers and probes (assays) were diluted (1:1) with Fluidigm GE assay loading reagent (Fluidigm; 100–7611). The assays and cDNA samples were loaded onto the Microfluidic chip as per the manufacturer’s instructions and 40 cycles of qPCR were performed on the BioMark Fluidigm Dynamic Array system.

### Binding antibody assay

Plasma antibody reactivity with 1086 C and 63521B Envs was determined via standard enzyme-linked immunosorbent assays (ELISA) and binding titers reported as log area under the curve^51,52^. In brief, 96-well plates were coated overnight at 4 °C with 15 μl of purified HIV-1 envelope proteins and blocked with assay diluent (PBS containing 4% whey protein, 15% normal goat serum, 0.5% Tween 20 and 0.05% sodium azide) for 2 h at room temperature. Plasma samples were added and the plate were incubated for 2 h at room temperature. Binding was detected with SureBlue Reserve TMB One Component microwell peroxidase substrate (KPL, Gaithersburg, MD). Development was stopped with 0.1 M HCl and plates were read at an optical density at 450 nm (OD450) with a VersaMax microplate reader (Molecular Devices, Sunnyvale, CA).

### ELISA for CXCL13

ELISA for CXCL13 were performed with plasma samples from pre-immunization, Week 2 and Week 32 time points from all monkeys in four groups using CXCL13 ELISA kit using manufacture’s protocol (R&D Systems, Minneapolis, MN).

### Neutralization assays

Neutralizing antibody titers were measured using a luciferase-based assay in TZM-bl cells^53,54^. This assay measures the reduction in luciferase reporter gene expression in TZM.bl cells following infection. In brief, threefold serial dilutions of plasma samples were performed in duplicate, and the 50% inhibitory concentration titer was calculated as the dilution that caused 50% reduction in relative luminescence units (RLU) compared with virus control wells after subtraction of cell control RLU. All data were analyzed using five-parameter curve fitting.

### Statistics

Mann–Whitney statistical tests were performed using GraphPad Prism Version 6.0.

### Reporting summary

Further information on research design is available in the Nature Research Reporting Summary linked to this article.

## Supplementary information

Supplementary Information

Reporting Summary

## Data Availability

The data that support the findings of this study are available from the corresponding authors upon request.
