# Supplementary material for: Recombinant MVA-prime elicits neutralizing antibody responses by inducing antigen-specific B cells in the germinal center
Source: NPJ Vaccines. 2021 Jan 25;6:15. doi: 10.1038/s41541-020-00277-1 (PMC7835239; doi:10.1038/s41541-020-00277-1)
Supplement: Supplementary file 1 — Supplementary Information [file 41541_2020_277_MOESM1_ESM.pdf]

Supplementary Table 1. Prime and boost immunization schedule

Group (n=6 per group)	Prime (Week)	Protein boost (Week)	Sample collection
Plasmid DNA	0, 4, 8	30	0, 10, 32
rMVA	0, 8	30	0, 10, 32
rVSV	0	30	0, 2, 32
Control	no prime	0, 10	0, 2, 12

Monkeys received intra-muscular immunization with vectors expressing Env C.1086. Primed monkeys received an MF59-adjuvanted rEnv C.1086 protein at week 30. Monkeys in Control group received two immunizations with rEnv C.1086 in combination with MF59 at weeks 0 and 10.

Supplementary Table 2. Analysis of fluidigm data with respect to vaccine group

rMVA vs. other vaccine groups	rMVA vs. rVSV	rMVA vs. DNA
IL4	TNFRSF11A RANK	CD4+84
CD4+27	ICOS	CSF1 MCSF
PTPN6 SHP1	PTPN6 SHP1	CD4+27
CD4+4	STAT1	CCR7
TBX21 TBET	IL6ST GP130	PTPN6 SHP1
BCL6	CD4+109	TNFSF10 TRAIL
IL13	IL4	RUNX1
CD4+84	BTLA	SMAD4
IL17	CD4+4	PRDM1 Blimp1
IL6ST GP130	LTB TNFc	FLIP
BAX	CD4+40LG	NKRF ITBA4
POU2AF1	RORA	GZMB
TGFBR3 BGCAN	IL13	TGFB1
CCL3	IL2RB	NFATC1
RUNX1	PDCD4+1	POU2AF1
CD4+69	LIF	IL10
IL10	TGFBR3 BGCAN	IL4
LTB TNFc	POU2AF1	MAF
MYC	NKRF ITBA4	IL2Rg CD4+132
CCR7	GZMA CTLA3	CD4+4

Supplementary Table 3. TaqMan Primers and Probes

Primer ID	Gene Symbol	Species
Hs00180269_m1	BAX	Human
Hs00277037_m1	BCL6	Human
Hs00154109_m1	BIRC3, CIAP2	Human
Hs00153353_m1	BIRC5	Human
Rh02889477_m1	BTLA	Rhesus
Rh02788104_gH	CCL3	Rhesus
Rh02788105_m1	CCL5	Rhesus
Hs00266213_s1	CCR3	Human
Rh02788181_s1	CCR6	Rhesus
Hs99999080_m1	CCR7	Human
Hs00370347_m1	CD109	Human
Rh02837950_m1	CD27	Rhesus
Hs00174796_m1	CD28	Human
Hs00181217_m1	CD4	Human
Rh02787995_m1	CD40LG	Rhesus
Hs00934033_m1	CD69	Human
Hs01547121_m1	CD84	Human
Hs01567025_m1	CD86	Human
Hs00233520_m1	CD8a	Human
Hs00192860_m1	CLEC2B, AICL	Human
Hs00174164_m1	CSF1, MCSF	Human
Rh02787987_m1	CTLA4	Rhesus
Hs00757930_m1	CXCL13, BLC	Human
Hs01847760_s1	CXCR3, MIGR	Human
Hs00540548_s1	CXCR5	Human
Hs00175210_m1	DPP4, CD26	Human
Hs00172872_m1	EOMES, TBR2	Human
Hs00531110_m1	FAS	Human
Hs00181225_m1	FASLG, CD95LG	Human
Hs01116280_m1	FLIP	Human
Hs00212860_m1	FOXP1	Human
Hs00203958_m1	FOXP3	Human
Hs01022023_m1	GABPA	Human
Rh02621745_g1	GAPDH	Rhesus
Hs00231122_m1	GATA3	Human
Hs00989184_m1	GZMA, CTLA3	Human
Hs01554355_m1	GZMB	Human
Hs00219575_m1	HLADRA	Human

Primer ID	Gene Symbol	Species
Rh02621771_m1	ICOS	Rhesus
Rh02788577_m1	IFNG	Rhesus
Rh02789325_m1	IL10	Rhesus
Hs01548202_m1	IL12RbII	Human
Rh03043053_m1	IL13	Rhesus
Hs00189606_m1	IL16	Human
Hs00174383_m1	IL17α	Human
Rh02789780_m1	IL2	Rhesus
Hs00222327_m1	IL21	Human
Hs00222310_m1	IL21R	Human
Hs00907777_m1	IL2RA	Human
Hs01081697_m1	IL2RB	Human
Hs00415671_m1	IL2Rg, CD132	Human
Rh02789319_m1	IL4	Rhesus
Hs00169842_m1	IL6R, CD126	Human
Hs00174360_m1	IL6ST, GP130	Human
Rh02841231_m1	IL7R	Rhesus
Rh02850395_m1	IRF4	Rhesus
Rh02841703_m1	LAG3	Rhesus
Hs01547250_m1	LEF1	Human
Hs00236874_m1	LTA, TNFb	Human
Hs00242739_m1	LTB, TNFc	Human
Hs00193519_m1	MAF	Human
Hs01032443_m1	MKI67, Ki67	Human
Hs00383033_m1	MTA3	Human
Rh02913645_m1	MYC	Rhesus
Hs00542678_m1	NFATC1	Human
Hs00213907_m1	NKRF, ITBA4	Human
Rh03418231_m1	PDCD1	Rhesus
Hs01573371_m1	POU2AF1	Human
Hs00153357_m1	PRDM1, Blimp1	Human
Hs00169359_m1	PTPN6, SHP1	Human
Rh00931151_m1	RORA	Rhesus
Rh02892670_m1	RORC	Rhesus
Hs00231079_m1	RUNX1	Human
Rh02929319_m1	RUNX3	Rhesus
Hs00158978_m1	SH2D1A, LYP	Human
Hs00232068_m1	SMAD4	Human

Primer ID	Gene Symbol	Species
Hs00705164_s1	SOCS1	Human
Rh02899274_m1	STAT1	Rhesus
Hs00598625_m1	STAT6	Human
Hs00894392_m1	TBX21, TBET	Human
Hs00998133_m1	TGFB1	Human
Hs00610318_m1	TGFBR1	Human
Hs01114253_m1	TGFBR3, BGCAN	Human
Hs99999139_m1	TIMP1	Human
Hs00174128_m1	TNF, TNFa	Human
Hs00187192_m1	TNFRSF11A, RANK	Human
Hs00533968_m1	TNFRSF4, OX40	Human
Hs00155512_m1	TNFRSF9, CD137	Human
Hs00921974_m1	TNFSF10, TRAIL	Human
Hs00198106_m1	TNFSF13B, BAFF	Human
Hs00184192_m1	TRAF2	Human
Hs00179626_m1	TRAT1, TRIM	Human
Hs99999081_m1	IL3	Human
Hs00929873_m1	CSF2	Human
Hs00234149_m1	SLAMF1, CD150	Human
Hs00171455_m1	LIF	Human

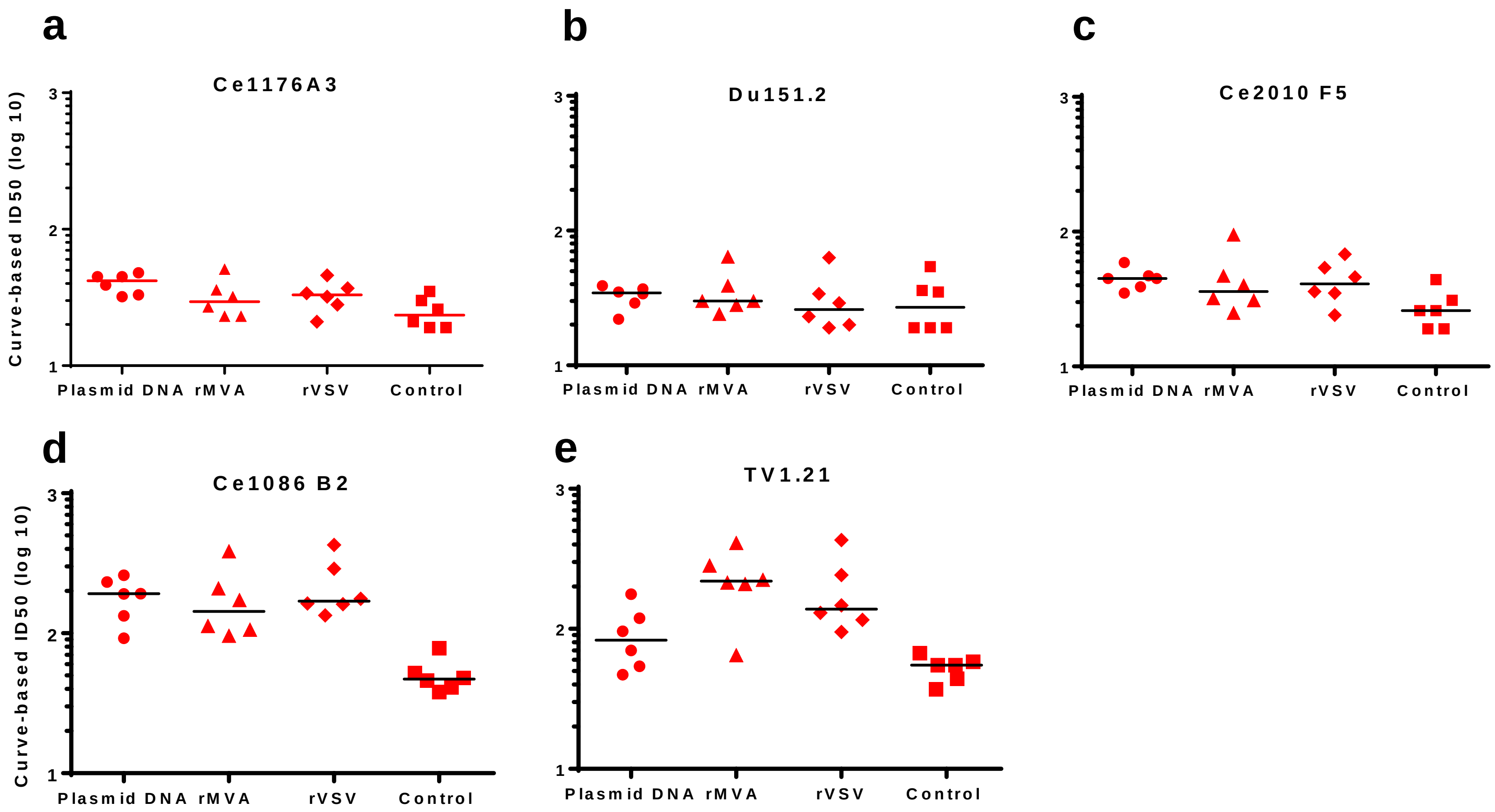

Supplementary Figure 1. Neutralizing antibody responses as measured by A3R5 cell-based assay. A3R5 cell-based neutralization assays were performed against replication-competent IMC.LucR tier 2 clade C viruses. Log ID50 values for pre-immunization (black symbols) and post-protein boost (red symbols) time points are shown for plasmid DNA (circles), rMVA (triangles) and rVSV (diamonds) groups. For protein only control group (squares) data from two weeks post second protein immunization at week 12 are shown. Panels a-e show ID50 titers against five different viruses, Ce1176 A3, Du151.2, Ce2010 F5, Ce.1086 and TV1.21. The line indicates the geometric mean of the six monkeys per group.

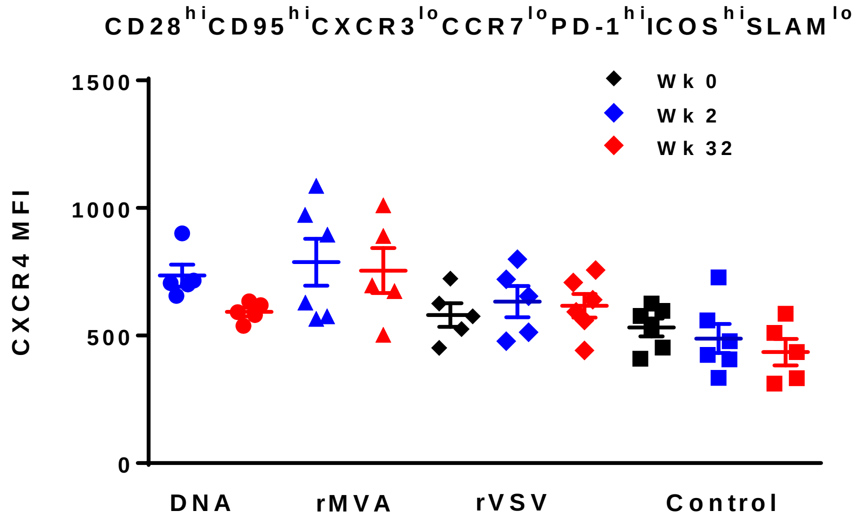

Supplementary Figure 2. CXCR4 level per cell as measured by Mean Fluorescence Intensity (MFI) are shown in lymph node-derived lymphocytes at indicated timepoints. Black symbols pre-priming; blue symbols post-priming and red symbols post-boost. Data from individual monkeys from four vaccine groups are shown. Bars depict mean $\pm$ SEM

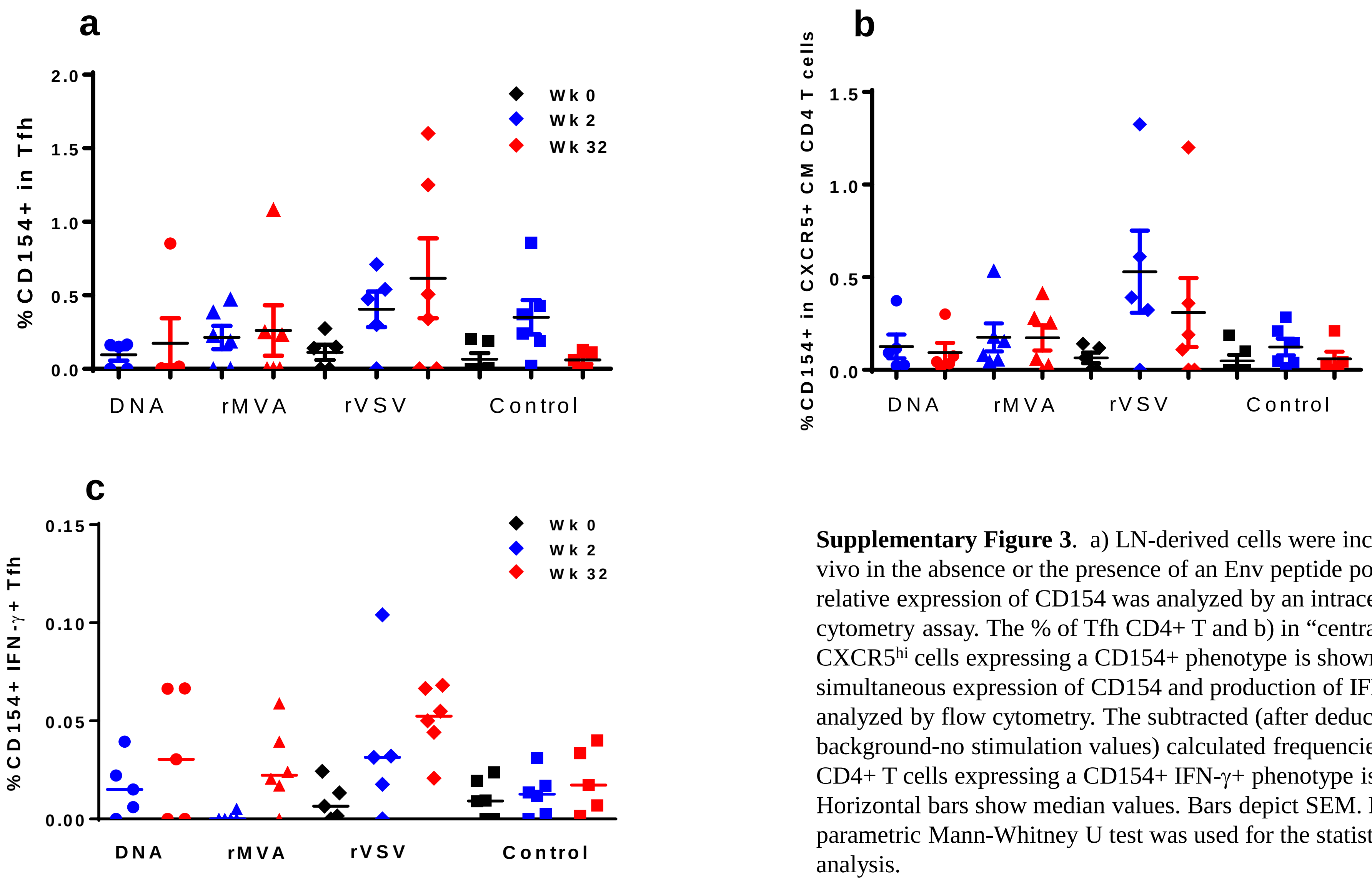

Supplementary Figure 3. a) LN-derived cells were incubated ex vivo in the absence or the presence of an Env peptide pool and the relative expression of CD154 was analyzed by an intracellular flow cytometry assay. The % of Tfh CD4+ T and b) in "central memory" $CXCR5^{hi}$ cells expressing a CD154+ phenotype is shown. c) The simultaneous expression of CD154 and production of IFN-γ was analyzed by flow cytometry. The subtracted (after deduction of the background-no stimulation values) calculated frequencies of Tfh CD4+ T cells expressing a CD154+ IFN-γ+ phenotype is shown. Horizontal bars show median values. Bars depict SEM. Non-parametric Mann-Whitney U test was used for the statistical analysis.

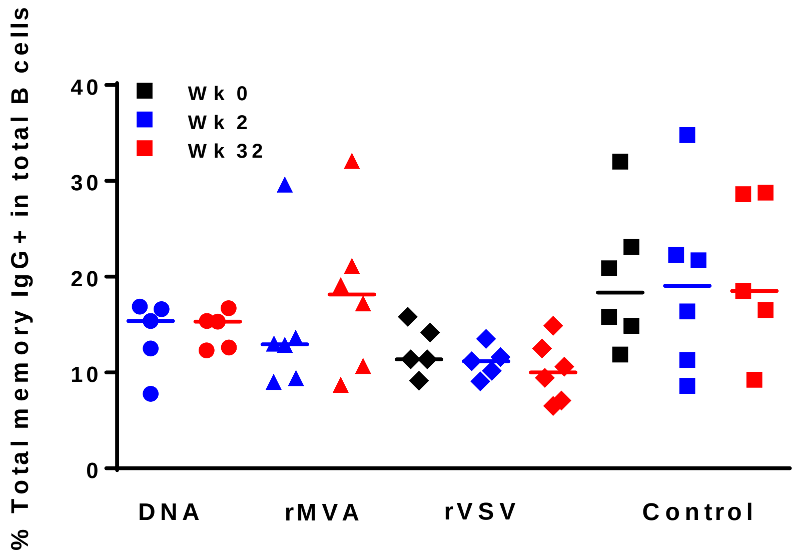

Supplementary Figure 4. Dot plot showing relative frequencies (%) of bulk B cells in LN tissues obtained at the indicated time points. Black symbols pre-priming; blue symbols post-priming and red symbols post-boost. The surface expression of IgG was analyzed by flow cytometry and the % of IgG+ bulk B cells from LN tissues is shown. The relative frequencies are shown as a percentage of total B cells. Horizontal bars show median values.

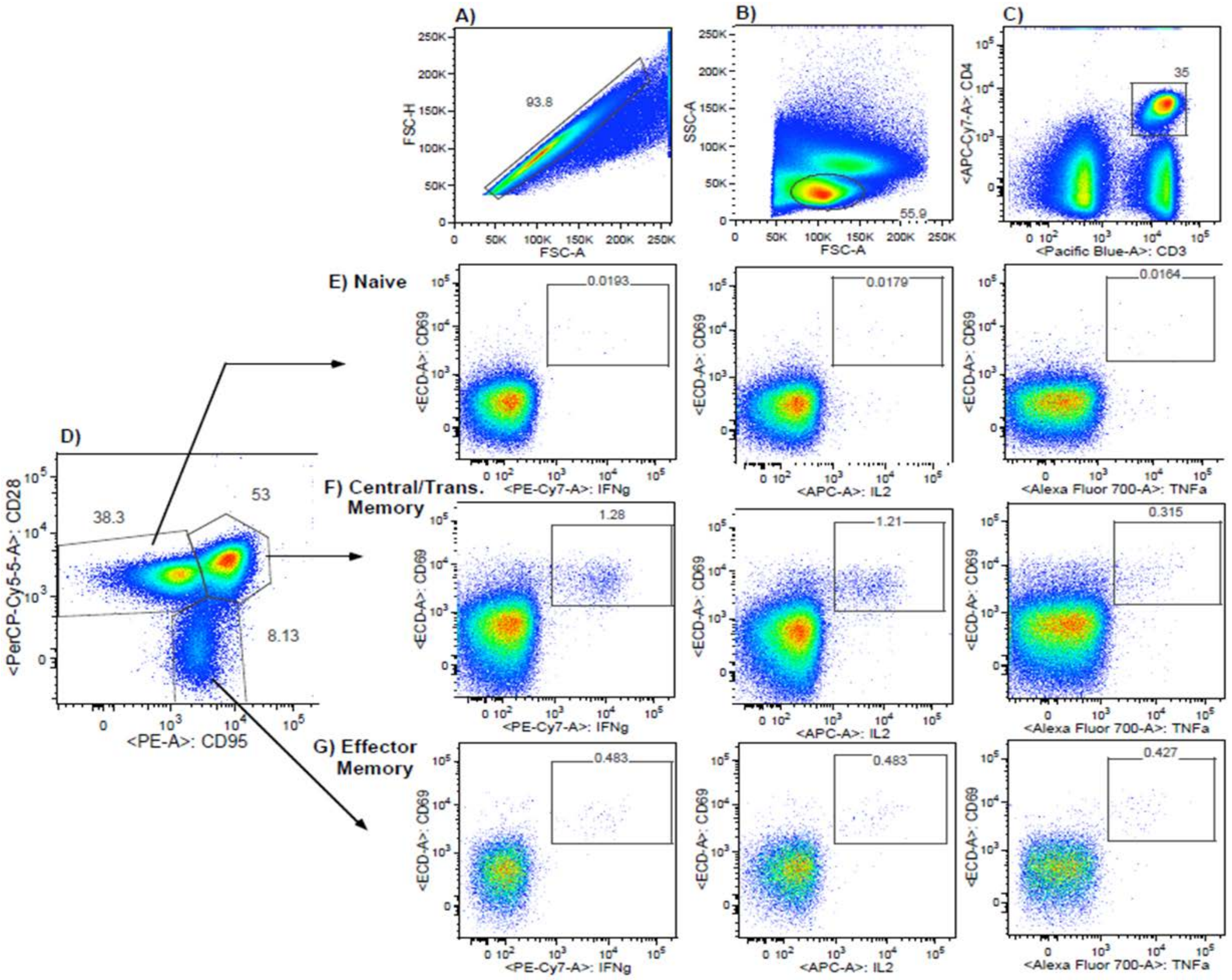

Supplementary Figure 5a Gating strategy for CD4+ naïve, effector memory and central/transitional memory T cells

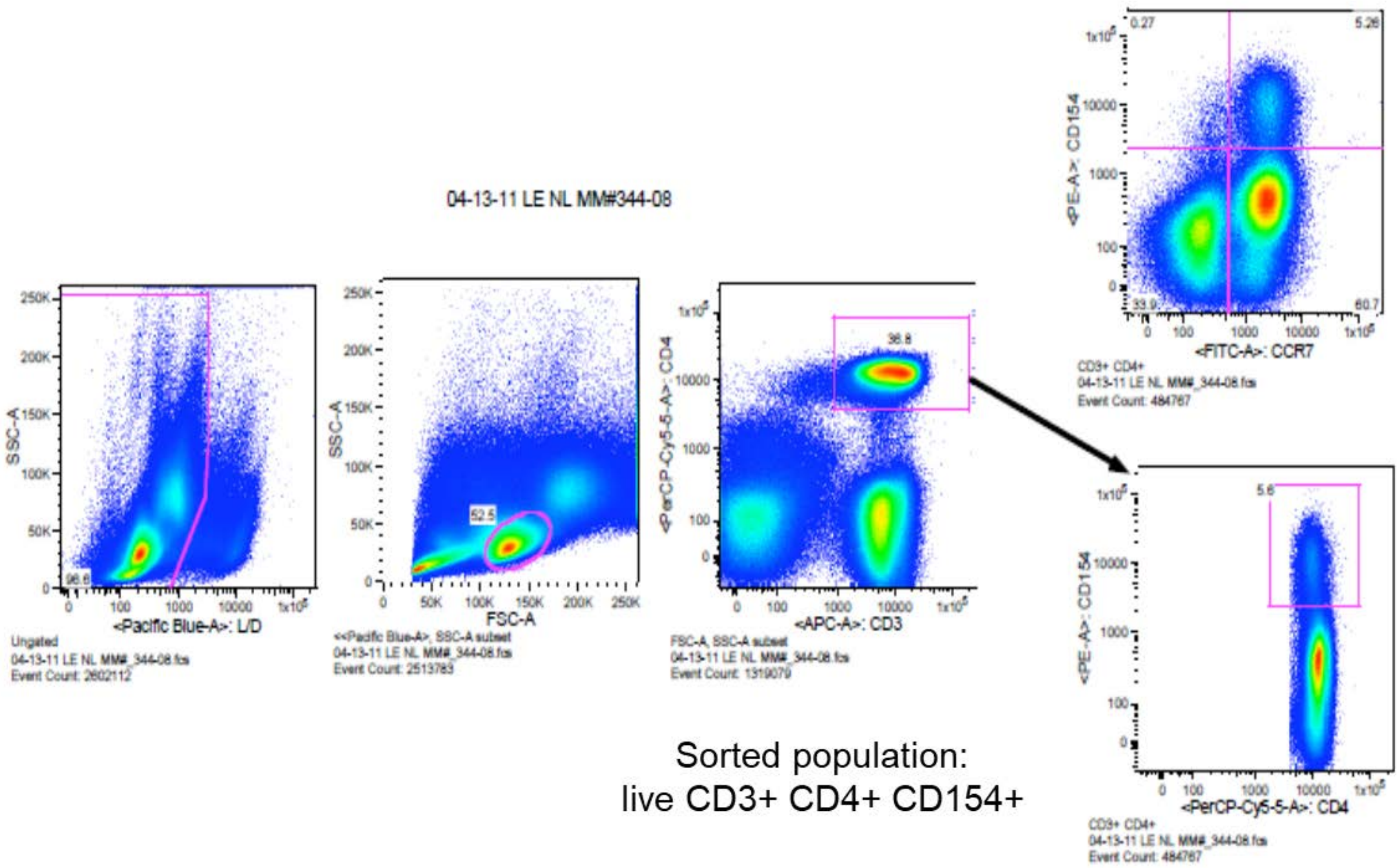

Supplementary Figure 5b Gating strategy for CD4+ CD154+T cell sort

c

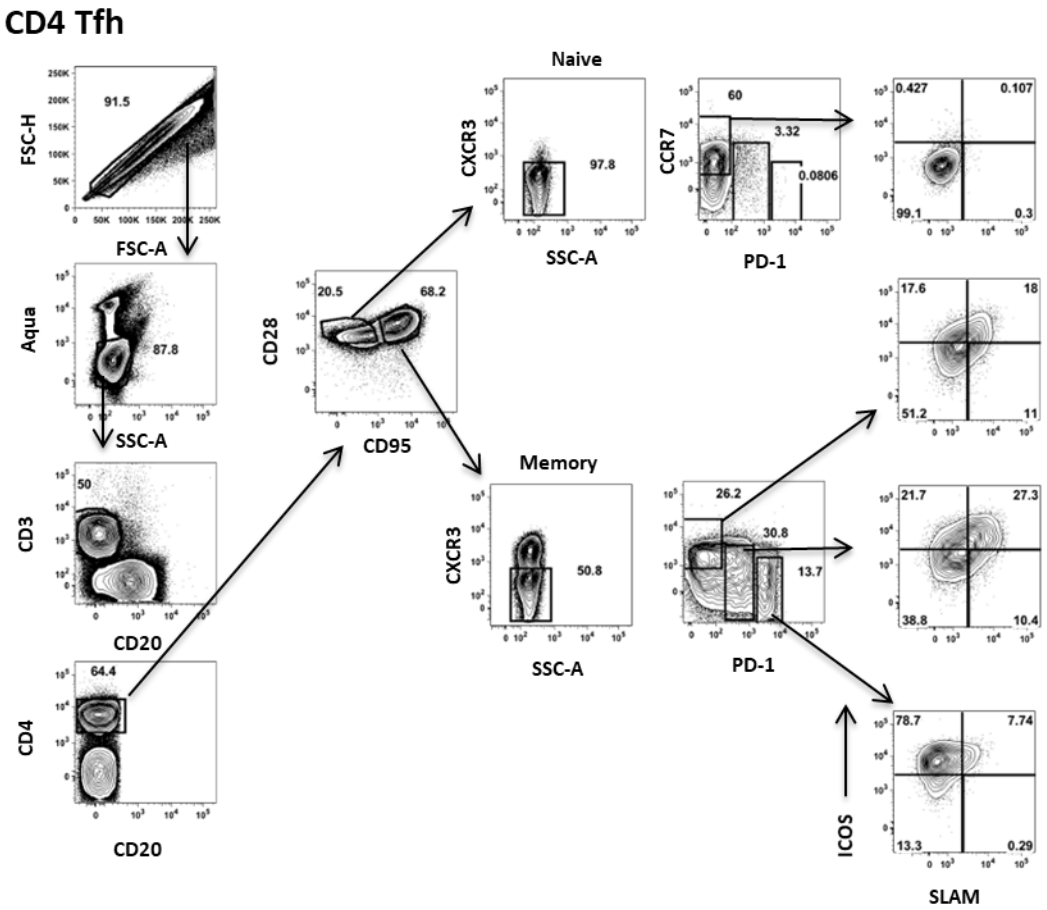

d

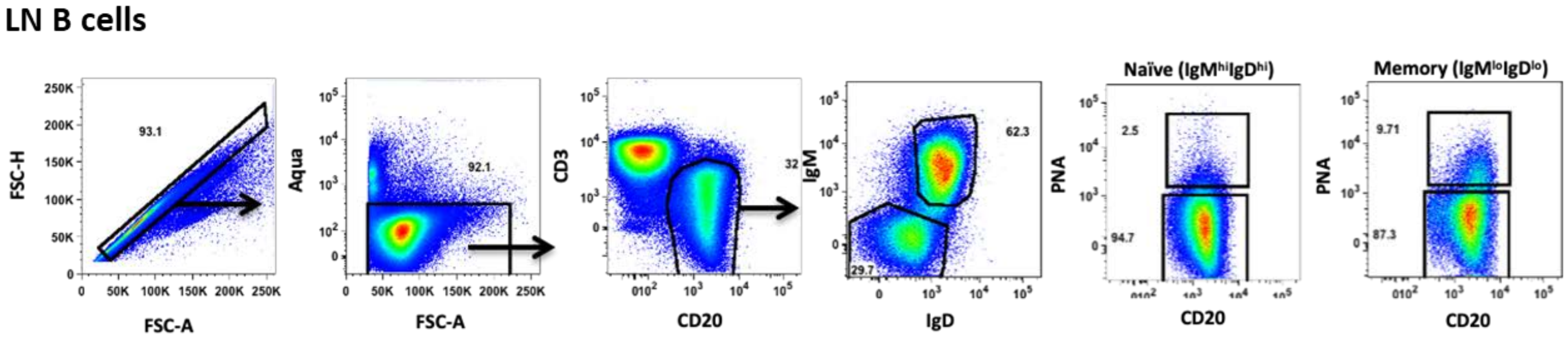

Supplementary Figure 5c-d Gating strategy for c) CD4+ Tfh cells d) lymph node B cells
