# Supplementary material for: Recombinant MVA-prime elicits neutralizing antibody responses by inducing antigen-specific B cells in the germinal center
Source: NPJ Vaccines. 2021 Jan 25;6:15. doi: 10.1038/s41541-020-00277-1 (PMC7835239; doi:10.1038/s41541-020-00277-1)
Supplement: Supplementary file 2 — Reporting Summary [file 41541_2020_277_MOESM2_ESM.pdf]

nature research

Corresponding author(s): Sampa Santra

Last updated by author(s): Oct 12, 2020

Reporting Summary

Nature Research wishes to improve the reproducibility of the work that we publish. This form provides structure for consistency and transparency in reporting. For further information on Nature Research policies, see our Editorial Policies and the Editorial Policy Checklist.

Statistics

For all statistical analyses, confirm that the following items are present in the figure legend, table legend, main text, or Methods section.

n/a	Confirmed	
☐	☒	The exact sample size (*n*) for each experimental group/condition, given as a discrete number and unit of measurement
☐	☒	A statement on whether measurements were taken from distinct samples or whether the same sample was measured repeatedly
☐	☒	The statistical test(s) used AND whether they are one- or two-sided<br>*Only common tests should be described solely by name; describe more complex techniques in the Methods section.*
☐	☒	A description of all covariates tested
☐	☒	A description of any assumptions or corrections, such as tests of normality and adjustment for multiple comparisons
☒	☐	A full description of the statistical parameters including central tendency (e.g. means) or other basic estimates (e.g. regression coefficient) AND variation (e.g. standard deviation) or associated estimates of uncertainty (e.g. confidence intervals)
☒	☐	For null hypothesis testing, the test statistic (e.g. *F*, *t*, *r*) with confidence intervals, effect sizes, degrees of freedom and *P* value noted<br>*Give P values as exact values whenever suitable.*
☒	☐	For Bayesian analysis, information on the choice of priors and Markov chain Monte Carlo settings
☒	☐	For hierarchical and complex designs, identification of the appropriate level for tests and full reporting of outcomes
☒	☐	Estimates of effect sizes (e.g. Cohen's *d*, Pearson's *r*), indicating how they were calculated

Our web collection on statistics for biologists contains articles on many of the points above.

Software and code

Policy information about availability of computer code

Data collection	BioMark Fluidigm Dynamic Array system; LSRII flow cytometer (BD Immunocytometry Systems)
Data analysis	FlowJo Version 9.6 (TreeStar, Ashland, OR).

For manuscripts utilizing custom algorithms or software that are central to the research but not yet described in published literature, software must be made available to editors and reviewers. We strongly encourage code deposition in a community repository (e.g. GitHub). See the Nature Research guidelines for submitting code & software for further information.

Data

Policy information about availability of data

All manuscripts must include a data availability statement. This statement should provide the following information, where applicable:
- Accession codes, unique identifiers, or web links for publicly available datasets
- A list of figures that have associated raw data
- A description of any restrictions on data availability

All figures have associated raw data and will be avilable

Field-specific reporting

Please select the one below that is the best fit for your research. If you are not sure, read the appropriate sections before making your selection.

☒ Life sciences ☐ Behavioural & social sciences ☐ Ecological, evolutionary & environmental sciences

For a reference copy of the document with all sections, see nature.com/documents/nr-reporting-summary-flat.pdf

Life sciences study design

All studies must disclose on these points even when the disclosure is negative.

Sample size	Sample size was determined upon consultation with a statistician
Data exclusions	Not applicable
Replication	Not applicable
Randomization	Not applicable
Blinding	Assay operators were blinded

Reporting for specific materials, systems and methods

We require information from authors about some types of materials, experimental systems and methods used in many studies. Here, indicate whether each material, system or method listed is relevant to your study. If you are not sure if a list item applies to your research, read the appropriate section before selecting a response.

Materials & experimental systems

n/a	Involved in the study	
☐	☒	Antibodies
☒	☐	Eukaryotic cell lines
☒	☐	Palaeontology and archaeology
☐	☒	Animals and other organisms
☒	☐	Human research participants
☒	☐	Clinical data
☒	☐	Dual use research of concern

Methods

n/a	Involved in the study	
☒	☐	ChIP-seq
☐	☒	Flow cytometry
☒	☐	MRI-based neuroimaging

Antibodies

Antibodies used	anti-CD4 APC-H7 (BD Biosciences), anti-CD95 PE (BD Biosciences), anti-CD28 PerCP-Cy5.5 (BD Biosciences;), and anti-CCR7 FITC (R&D Systems). After fixation and permeabilization with Cytofix/Cytoperm solution (BD Biosciences), the cells were stained with anti-IFNγ PECy7 (BD Biosciences), anti-TNFα AF700 (BD Biosciences), anti-IL2 APC (BD Biosciences), anti-CD3 Pacific Blue (BD Biosciences), and anti-CD69 ECD4+ (Beckman Coulter
Validation	Validation of each antibody is performed prior to use by the flow cytometry core

Animals and other organisms

Policy information about studies involving animals; ARRIVE guidelines recommended for reporting animal research

Laboratory animals	Macaca mulatta (rhesus monkey), both male and female
Wild animals	N/A
Field-collected samples	N/A
Ethics oversight	Guide for the Care and Use of Laboratory Animals and Institutional Animal Care and Use Committee of Harvard Medical School

Note that full information on the approval of the study protocol must also be provided in the manuscript.

Flow Cytometry

Plots

Confirm that:

☐ The axis labels state the marker and fluorochrome used (e.g. CD4-FITC).

☐ The axis scales are clearly visible. Include numbers along axes only for bottom left plot of group (a 'group' is an analysis of identical markers).

☐ All plots are contour plots with outliers or pseudocolor plots.

☒ A numerical value for number of cells or percentage (with statistics) is provided.

Methodology

Sample preparation	Peripheral blood mononuclear cells (PBMC) were incubated in RPMI supplemented with 10% fetal bovine serum, 100U/ml penicillin and 100 ?g/ml streptomycin (RPMI-10) at 37ºC in a 5% CO_2 environment for 6 hours in the presence of either DMSO (unstimulated), pool of overlapping C.1086 Env peptides or 0.4µg/well staphylococcal enterotoxin B (SEB) (Sigma) as positive control. The peptide pool spanning the entire HIV-1 Env C.1086 comprised of 15 amino acid peptides overlapping by 11 amino acids. All wells contained 2µM of a protein transport inhibitor, monensin (GolgiStop; BD Biosciences) and 1µg/ml anti-human CD49d antibody (BD Biosciences). The cultured cells were then stained with the following mAbs: anti-CD4 APC-H7 (BD Biosciences), anti-CD95 PE (BD Biosciences), anti-CD28 PerCP-Cy5.5 (BD Biosciences;), and anti-CCR7 FITC (R&D Systems). After fixation and permeabilization with Cytofix/Cytoperm solution (BD Biosciences), the cells were stained with anti-IFNγ PECy7 (BD Biosciences), anti-TNFα AF700 (BD Biosciences), anti-IL2 APC (BD Biosciences), anti-CD3 Pacific Blue (BD Biosciences), and anti-CD69 ECD4+ (Beckman Coulter,), and fixed with 1% formaldehyde.
Instrument	LSR II flow cytometer (BD Biosciences)
Software	LSR II flow cytometer (BD Biosciences)
Cell population abundance	*Describe the abundance of the relevant cell populations within post-sort fractions, providing details on the purity of the samples and how it was determined.*
Gating strategy	*Describe the gating strategy used for all relevant experiments, specifying the preliminary FSC/SSC gates of the starting cell population, indicating where boundaries between "positive" and "negative" staining cell populations are defined.*

☐ Tick this box to confirm that a figure exemplifying the gating strategy is provided in the Supplementary Information.
